# Compliance to iron folic acid supplementation and its associated factors among pregnant women attending Antenatal clinic in Wondo district: a cross-sectional study

**DOI:** 10.1038/s41598-023-44577-7

**Published:** 2023-10-14

**Authors:** Taye Mengistu, Bikila Lencha, Ashenafi Mekonnen, Sisay Degno, Daniel Yohannis, Girma Beressa

**Affiliations:** 1 Wondo District Health Office, Wondo West Arsi Zone, Oromia, Ethiopia; 2 Department of Public Health, Madda Walabu University, Shashemene, Oromia Ethiopia; 3 Department of Midwifery, Madda Walabu University, Shashemene, Oromia Ethiopia; 4 Department of Public Health, Madda Walabu University, Bale Goba, Oromia Ethiopia

**Keywords:** Health care, Risk factors

## Abstract

Pregnant women are at high risk for iron deficiency anemia due to increased nutrient requirements during pregnancy. Despite high coverage of iron and folic acid supplementation (IFAS), low compliance is reported. The study aimed to assess compliance with IFAS and its associated factors among antenatal care (ANC) attendees in Wondo District, Southern Ethiopia. A facility-based cross-sectional study was conducted among 400 pregnant women. Pregnant women were selected through systematic random sampling. Pre-tested structured questionnaire was used to collect data through face to face interview. Data were entered into Epi-info and exported to Statistical Package for Social Sciences for analysis. The variables with p-value < 0.25 in the bivariable analysis were entered into the multivariable logistic regression model. P values less than 0.05 were considered significant. Results were reported as crude and adjusted odds ratios with 95% confidence intervals. The prevalence of compliance to IFAS was (177, 44.3%). Factors significantly associated with compliance to IFAS were maternal age ≥ 25 years [AOR 2.27, 95% CI (1.21, 4.28)], maternal education [AOR 2.62, 95% CI (1.43, 4. 79)], husband's education [AOR 3.60, 95% CI (2.07, 6.25)], knowledge of anemia [AOR 4.40, 95% CI (2.65, 7.30)], and knowledge of IFA [AOR 2.21, 95% CI (1.40, 3.50)]. This study showed that compliance to IFAS was low. Maternal age, maternal education, husband's education, knowledge about anemia and iron folic acid was found to be significantly associated with adherence to IFAS. Emphasis should be placed on young, uneducated mothers and their husbands.

## Introduction

Compliance refers to the act of conforming to the recommendations made by the provider with respect to timing, dosage, and frequency of medication taking. Therefore, compliance is the extent to which a client acts in accordance with the prescribed interval and dose of a dosing regimen^1^. Anemia is a condition in which the oxygen-carrying capacity of red blood cells is insufficient to meet the physiological needs of the body^2^. It is a global public health problem affecting nearly two billion people, despite the global introduction of iron and folic acid and their provision through antenatal care programs^3^. It affects more than 32 million pregnant women worldwide and contributes to 20% of maternal deaths^4^ Iron deficiency is the most common and widespread nutritional disorder in the world and is a major public health problem in both developed and developing countries^5^. Pregnant women in particular are at high risk for iron deficiency anemia due to their high nutrient requirements during pregnancy^6^. Preventive measures include daily iron and folic acid supplementation to reduce the risk of low birth weight, neural tube defects, and maternal anemia and iron deficiency during pregnancy^7^.

Globally, an estimated 41.8% of pregnant women are anemic, and at least half of this burden is thought to be due to iron deficiency, with the remainder due to conditions such as folate, vitamin B12 or vitamin A deficiency, chronic inflammation, parasitic infections and inherited disorders^7^. Iron deficiency anemia during pregnancy is associated with an increased risk of low maternal weight gain, preterm labor and placenta previa, premature rupture of membranes, cardiac arrest and bleeding, decreased resistance to infection, poor cognitive development, and reduced work capacity^6,8^. Similarly, the effects of iron deficiency anemia on the fetus and newborn are increased risk of prematurity, low birth weight, and fetal distress, which contribute to perinatal morbidity and mortality^6,9–11^.

Iron supplementation has been a major strategy in low and middle-income countries including Ethiopia to reduce iron deficiency anemia during pregnancy^12^. In Ethiopia, IFA is supplemented free of charge during antenatal care follow up. Despite the implementation of IFAS in Ethiopia, anemia remains responsible for the majority of maternal morbidity and mortality^13^. Therefore, compliance to IFAS is critically needed. Compliance to iron and folic acid supplementation (IFAS) is defined based on the woman's self-report of the number of IFA tablets taken in the previous 7 days, which was used as a proxy estimate of recommended (90 + days) iron folic acid compliance. In this case, pregnant mothers who took ≥ 4 tablets per week were considered compliant^14^.

National Nutrition Program II is being designed and implemented in Ethiopia to prevent and treat anemia during pregnancy^15^. This program aims to reduce the prevalence of anemia in pregnant women from 22 to 14% by 2020. This goal includes providing iron sulfate and folic acid to all pregnant women^16^. According to the national standard, the recommended dose in Ethiopia is 300–325 mg of ferrous sulfate and 400 µg of folic acid once a day for at least 90 days of prenatal period, preferably with a meal^12^. But as the national data and most studies show, compliance is still below the standard. For example, the 2019 Ethiopia Mini Demographic Health Survey (EMDHS) report found that among 3,927 pregnant women in the past five years, 60% took iron folic acid (IFA) tablets during pregnancy, and 11% took them for the recommended period of 90 or more days^17^.

Recent studies conducted in Ethiopia have reported prevalence of IFAS compliance ranging from 38.3% in Shala District^18^ to 92.4% in Robe town Southeast Ethiopia^19^. However, the Kanungu District study in Uganda reported the lowest prevalence of 22.37% compared to the most recent studies conducted in Ethiopia^20^. This shows the variation from one location to another in Ethiopia and the huge differences from country to country, which necessitates site-specific prevalence studies.

Previous studies have shown that education, maternal age, family income, place of residence, early initiation of ANC, frequency of ANC, history of anemia, counseling for IFAS during pregnancy, knowledge of anemia, and knowledge of IFAS were associated with adherence compliance to IFAS^8,21–28^. However, recent studies conducted in Ethiopia and Uganda found no significant association between sociodemographic variables and IFAS adherence^18,20,29,30^. Nevertheless, socio-demographic variables are important determinants of compliance to IFAS as shown in the previous studies.

According to the Wondo District Health Office statistics report, the level of iron and folic acid supplementation among pregnant women in the district was 99%. However, the level of compliance with IFA in the district has not yet been assessed. Determining the level of compliance with IFA and identifying the factors responsible for compliance in the district could make a significant contribution to improving the health status of pregnant women and newborns. Therefore, this study aimed to assess the compliance with IFAS and its associated factors among pregnant women in Wondo District of Oromia, Southern Ethiopia.

## Methods

### Study setting, period and participants

A facility-based cross-sectional study was conducted among pregnant women in Wondo District, Southern Ethiopia. According to district health office administrative report, the total number of women of reproductive age groups and pregnant women in the district were estimated to be 26,183 and 4,105 respectively. Among pregnant women attending ANC clinic at health centers, 97% were supplemented with IFA tablet^31^. The study was conducted from August 16 to October 30, 2019.

All pregnant women attending ANC and previously supplemented with iron and folic acid tablets at the facilities at least one week prior to the data collection period were our source population. Pregnant women who were previously supplemented with iron and folic acid tablets at least one week prior to data collection, who came to the health facility during the data collection period, were systematically selected for the study population.

#### Inclusion and exclusion criteria

Inclusion criteria for this study were pregnant women who had at least one ANC visit at a health facility and were supplemented with IFA tablets at least one week prior to data collection.

Pregnant women who came for the first antenatal visit, those who could not hear and/or speak and those with mental disorder were excluded from the study.

### Sample size determination and Sampling procedure

The minimum sample size required was calculated using the single population proportion formula, assuming 95% CI, 5% margin of error, and a proportion of 39.2% from the study in Misha district of Hadiya zone^23^. Finally, considering 10% for possible non-response, a sample size of 402 was estimated to meet the objectives.

The study was conducted in three health centers namely Busa, Shasha and Bachil located in Wondo district. Out of 853 pregnant mothers attending ANC clinic in the three health centers, 830 (97%) were supplemented with IFA within two months and fifteen days. The calculated sample was distributed among these health centers in proportion to the number of women attending ANC and supplemented IFA during the data collection period. Finally, systematic random sampling technique was used to include 402 participants in the study using the interval (K = 2) for each health center. Lottery method was used to select the random starting number and 1 was drawn and odd numbers of participants were interviewed after receiving ANC service.

### Data collection

Data were collected using a structured questionnaire adapted from a study conducted in Dabre Tabor General Hospital^32^. It consisted of six parts: socio-demographic characteristics of participants, pregnancy and health status characteristics, knowledge of IFA supplementation, compliance question, knowledge of anemia, health facility related factor (S2). Knowledge questions were checked for reliability and the result of Cronbach's alpha was equal to 0.7.

#### Measurement of variables

Our dependent variable for the current study was IFAS compliance, which is a dichotomous variable.

##### Compliance

This was measured based on the woman's self-report of the number of IFA tablets taken in the previous 7 days, which was used as a proxy estimate of recommended (90 + days) iron folic acid compliance. In this case, pregnant mothers who took ≥ 4 tablets per week were considered compliant^14^.

##### Knowledge of anemia

Women's knowledge of anemia was assessed by five multiple-choice questions asking about signs and symptoms of anemia, possible causes of anemia, groups susceptible to anemia, and prevention of anemia. The correct answer for each item was scored as "1" and the incorrect answer was scored as "0". The items were then summed and the mean was calculated. If the pregnant women scored above the mean, they have good knowledge and if the pregnant women scored below the mean, they have poor knowledge^27^.

##### Knowledge of IFAs

Women's knowledge of IFA supplementation was assessed by four multiple-choice questions asking about the benefits of IFA, frequency of use, duration of use, and side effects. The correct answer for each item was scored as "1" and the incorrect answer was scored as "0". The items were then summed and the mean was calculated. If the pregnant women scored above the mean, they had good knowledge and if the pregnant women scored below the mean, they had poor knowledge^27^.

### Data analysis

Data were checked for completeness and consistency, coded and entered into EPI-info version 7.2 and exported to SPSS version 21 for analysis. Univariate analysis was performed for each variable by describing frequencies (percentages) for categorical variables and means (standard deviation) for continuous variables. Binary logistic regression analysis was performed for each independent variable with the outcome variable. A variable with a p-value < 0.25 in the bivariate test was considered a candidate for multivariable logistic regression analysis. Finally, a multivariable logistic regression model was performed to determine the independent variables of IFA compliance status. Adjusted odds ratios with corresponding 95% confidence intervals were calculated to determine the strength of the association. All tests were two-tailed and a p-value < 0.05 was considered statistically significant.

### Ethics approval and consent to participate

Prior to the actual data collection process, the Madda Walabu University Ethical Review Committee approved the study protocol with reference number MWU/950/12 E.C. Additional written permission was obtained from the West Arsi Zonal Health Office and the Wondo District Health Office on a step-by-step basis. Subjects were informed of the purpose of the study and were assured of privacy and confidentiality of their responses. Written informed consent was obtained from all the participants and or their legal guardians. Assent was obtained from women under 18 years of age, and their husbands were asked for consent. The study adhered to the tenets of the Declaration of Helsinki.

## Results

### Socio-demographic characteristics

A total of 400 participants were involved in the study with a response rate of 99%. The reason for non-response was refusal. The age of the study participants ranged from 16 to 43 years with a mean age of 28.68 (± 5.89 SD) years. Most had no formal education (185, 46.2%), lived in rural areas (301, 75.3%), and were Protestant in religion (183, 45.8%). Almost three quarters (296, 74.0%) of the study participants had more than 6 family members (Table 1).

**Table 1:** Socio-economic and demographic characteristic of the respondents in Wondo district, Oromia, Southern Ethiopia, August 2019.

Variable	Frequency	Percent
Age		
15–19	31	7.8
20–24	52	13.0
> = 25	317	79.2
Religion		
Orthodox	97	24.2
Protestant	183	45.8
Muslim	120	30.0
Place of residence		
Urban	99	24.8
Rural	301	75.2
Marital status		
Married	390	97.5
Divorce	7	1.8
Widowed	3	0.8
Maternal education		
No formal education	185	46.3
Primary	92	23
Secondary and above	123	30.8
Husband education		
No formal education	219	54.8
Primary	99	24.8
Secondary and above	82	20.4

### Obstetrics and health related characteristics

Among the respondents, (220, 55%) were tested for anemia and (48, 21.8%) of them were anemic during the current pregnancy. Most of the pregnant women, (249, 62.2%) visited antenatal care follow-up for 3–4 times, while (151, 37.8%) had 2 antenatal care visits in the health institution during the current pregnancy.

### Knowledge status of respondents on Anemia and Benefit of IFAS

**Figure 1:**
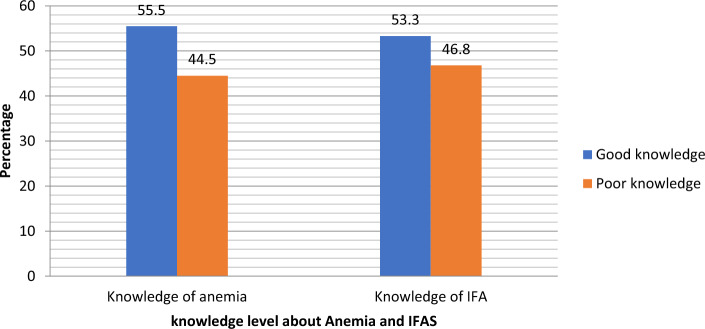
Anemia and IFA Knowledge related characteristics of study participants. The figure shows the knowledge of anemia and knowledge of IFAS benefits.

About (222, 55.5%) of the respondents had good knowledge about anemia whereas (178, 44.5%) had poor knowledge about anemia. Regarding the knowledge about the benefits of IFAS, (213, 53.2%) of the respondents had good knowledge while (187, 46.8%) had poor knowledge about IFAS. (Fig. 1).

#### Health facility related characteristics

More than half (210, 52.5%) were counseled on IFA during their visits. The majority (222, 55.5%) of respondents needed 30 min or less to reach the nearest health facility from their home (Table 2).

**Table 2:** Health facility related characteristics of pregnant women attending ANC clinic in Wondo district, Oromia, 2019.

Variable	Frequency	Percent
Counseled on IFA		
Yes	210	52.5
No	190	47.5
Time to reach Health Facility in minute		
> 30	178	44.5
≤ 30	222	55.5
Time spent in the facility in minute		
> 30	117	29.2
≤ 30	283	70.8

### Compliance status to IFA supplementation

From the total of 400 participants enrolled in this study, (117, 44.3%) with 95% CI of (39.0%, 50.0%) of the pregnant women were compliant with IFA, that is, took IFA tablets for ≥ 4 days/week in the previous one week before the study.

### Factors associated with compliance to IFA among pregnant women

Binary and multivariable logistic regression was done to identify factors associated with compliance to IFA supplementation. First, sixteen factors were analyzed by using binary logistic regression. Among them, maternal age ≥ 25 years [COR 2.47, 95% CI (1.45,4.18)], maternal primary education [COR 1.91, 95% CI (1.50,3.16)], husband primary education [COR 2. 77, 95% CI (1.70,4.52)], husband's secondary education and above [COR 2.38, 95% CI (1.42,3.99)], counseling on IFA [COR 1.28, 95% CI (0.86,1.90)], knowledge of IFA [COR 2. 08 95% CI (1.39,3.12)], knowledge of anemia [COR 3.2795% CI (2.15,4.97)], gestational age at 1st ANC [COR 1.28, 95% CI (0.87,1.91)], number of ANC visits [COR 1. 60, 95% CI (1.06,2.42)] and time from home to health facility [COR 1.27, 95% CI (0.85,1.89)] had p-value < 0.25 and were selected for multivariable analysis.

Multivariable analysis revealed that five predictors were significantly associated with IFA supplementation. Pregnant mothers whose age was ≥ 25 years were 2.27 times more likely to adhere to IFAS than pregnant mothers whose age was < 25 years (AOR 2.27, 95% CI (1.21, 4.28)). The odds of compliance to IFAS were 2.62 times higher among participants whose educational status was primary education [AOR 2.62, 95% CI (1.43, 4.79)] compared to study participants who were illiterate. And those study participants whose husbands had an educational status of primary education were 3.60 [AOR 3.60, 95% CI (2.07, 6.25)] and secondary education and above were 2.52 [AOR 2.52, 95% CI (1.40, 4.52)] times more likely to comply with IFAS as compared to those study subjects whose husbands had no formal education. Pregnant mothers who had good knowledge about IFA supplementation were 2.21 times more likely to be compliant with IFA supplementation as compared to those who had poor knowledge about IFA supplementation (AOR 2.21, 95% CI (1.40, 3.50)). Pregnant mothers who had good knowledge about anemia were 4.40 times more likely to comply with IFA supplementation compared to those who had poor knowledge (AOR 4.40, 95% CI (2.65, 7.30)) (Table 3).

**Table 3:** Factors associated with compliance to IFAS among Pregnant women in Wondo District, Oromia, Ethiopia, 2019.

Variable	Compliance	Noncompliance	COR (95%CI)	AOR (95%CI)
Age				
< 25	23	60	Ref	Ref
> = 25	154	163	2.47(1.45,4.18)	2.27(1.21,4.28)*
Maternal education				
No formal education	75	110	Ref	Ref
Primary	52	40	1.91(1.50,3.16)	2.62(1.43,4.79)*
Secondary and above	50	73	1.01(0.63,1.60)	0.57(0.34,1.02)
Husband education				
No formal education	74	145	Ref	Ref
Primary	58	41	2.77(1.70,4.52)	3.60(2.07,6.25)*
Secondary and above	45	37	2.38(1.42,3.99)	2.52(1.40,4.52)*
Counseled on IFA				
No	78	112	Ref	Ref
Yes	99	111	1.28(0.86,1.90)	1.28(0.81,2.02)
Knowledge of anemia				
Poor knowledge	51	127	Ref	Ref
Good knowledge	126	96	3.27(2.15,4.97)	4.40(2.65,4.52)*
Knowledge of IFA				
Poor knowledge	65	122	Ref	Ref
Good knowledge	112	101	2.08(1.39,3.12)	2.21(1.40,3.50)*
Time of first ANC				
> 16 weeks	68	99	Ref	Ref
< = 16 weeks	109	124	1.28(0.87,1.91)	1.49(0.87,2.55)
Number of ANC visit				
2 visits	56	95	Ref	Ref
3–4 visits	121	128	1.60(1.06,2.42)	0.80(0.50,1.28)
Time to reach HF				
> 30	73	105	Ref	Ref
< = 30	104	118	1.27(0.85,1.89)	1.14(0.73,1.81)

*IFA* iron and folic acid, *ANC* ante natal care, *Ref* reference, *COR* crude odds ratio, *AOR* adjusted odds ratio, *CI* confidence interval, *HF* health facility.

*Statistical significance at p < 0.05.

## Discussion

This study was conducted to assess compliance with IFAS and identify factors associated with it among pregnant women in Wondo District of Oromia Region. Among 400 pregnant women who participated in the study, (177, 44.3%) were compliant to IFA supplementation. This finding is higher than the findings of the study conducted in Mecha District, Northwest Ethiopia (20.4%)^27^, Afar Region, Mille and Assaita Districts 22.9%^33^, Shalla district 38.3%^18^, Lay Armachiho Health Centers (28.5%)^22^ and the study conducted in Kanungu District, Uganda (22.37%)^20^. This may be related to increased knowledge of pregnant women about anemia and IFA supplementation over time. The educational level of the study participants and their husbands may also have increased compliance in our study. On the other hand, the result of compliance with IFA found in this study was lower than the result found in Assella city which was 59.8%, which was 59.8%^16^ , the study in Burji district Segen zone 51.4%^14^, Zinder 68%^8^, Simada district , North West Ethiopia (67.6%)^29^, Debay Tilat Gen district North West Ethiopia (52.8)^34^, Dire Dawa city (71.8%)^30^and the study done in South India 64.7%^35^. This inconsistency may be due to cultural, socioeconomic, and geographic differences from place to place. For example, in the Diredawa study, slightly less than half of the participants were government employees, and being an employee may have increased their awareness of IFA^30^. Another reason may be that the majority of our study participants were from rural areas and pregnant women may not receive information from the health center, and women in rural areas were probably less educated than those in urban areas. The timing of the study is another possible reason for the observed difference.

This study revealed that maternal educational status had a significant association with compliance to IFAS. Pregnant women with primary education were 2.62 times more likely to adhere to IFAS than pregnant women with no formal education. This finding is supported by other studies conducted in Mecha District, North west Ethiopia, Nigeria^21,22^ and Indonesia^21,26,27,36^. This is also similar to the study from the Philippines, where better education led to compliance with IFAS^37^. This may be because educated women are likely to have better knowledge and access to information about iron deficiency anemia and its treatment, the benefits of supplements, and pregnancy in general. Again, it may be related to the notion that education is more likely to increase women's awareness of micronutrient deficiencies and ways to overcome them. It may also be related to the fact that educated women are more likely to adhere to health care interventions, such as IFA, that provide better care for both the infant and the mother.

Participant`s husband`s educational status was also one of the significantly associated factor, those participant`s whose husband`s educational status was primary level were 3.60 times, secondary level and above were 2.52 times more compliant than those participant`s whose husband had no formal education. This finding is consistent with the study conducted in Indonesia^36^. The likely explanation could be that partner support from educated partners may have helped women to adhere to IFAS. Educated husbands may have been aware of IFAS and encouraged their partners to take the tablets without interruption as recommended by health workers. Encouragement from husbands may thus motivate women to continue taking the tablets.

Women aged ≥ 25 years were 2.27 times more likely to be compliant with IFAS than women of younger age (< 25 years). The reason for this may be that older women may be more concerned about their health and pregnancy outcomes and may have had better experiences with prevention and treatment of iron deficiency anemia. This finding is consistent with a study in India that found that older women (> 25 years) were more compliant than younger and this finding is also consistent with the study in Ethiopia^23^. However, some recent studies conducted in Ethiopia found no significant association between socio-demographic variables (age, mother's education, and in our case, husband's education) and IFAS adherence^18,29,30,34^.

Good knowledge of anemia was significantly associated with compliance to IFAS among pregnant women, where pregnant women with good knowledge of anemia were 4.40 times more likely to adhere to IFAS than those with poor knowledge of anemia This finding is similar with the studies conducted in North Wollo zone^24^, Western Amhara, Mecha district^27^ and Dire Dawa city^30^. The probable reason could be the fact that knowledge helps women to have awareness about prevention and treatment of anemia by taking iron folate supplement during pregnancy.

Knowledge of IFAS was also associated with women's adherence to iron and folic acid supplementation in studies conducted in Kenya, Hawasa City, Dire Dawa City, Debay Tilat Gen District North West Ethiopia, and Debre tabor General Hospital^6,30,32,34,38^. Similarly, knowledge of iron and folic acid supplementation was associated with adherence to iron and folic acid supplementation in this study. This may be because knowledge helps women understand the benefits of taking the supplement and the consequences of not taking the supplement for the mother and fetus during pregnancy, labor and delivery.

The strength of this study is that we used a relatively large sample and the response rate was high. However, the study is prone to social desirability bias because the outcome variable depends on mothers' self-report. This may underestimate or overestimate compliance. In addition, recall bias is another concern. We tried to minimize this by collecting information on reported IFA intake within 7 days prior to the interview. Despite the limitations, the current study identified valuable variables for intervention.

## Conclusions

The results of this study showed that pregnant mothers' compliance to IFAS was 44.3%, and factors such as maternal age, maternal education, husband's education, knowledge of anemia, and knowledge of IFAS were found to be significantly associated with compliance to IFAS during pregnancy. Compliance to prenatal iron and folic acid is still low in the district and does not yet meet WHO recommendations, despite the usefulness of this health program to combat iron deficiency anemia. Therefore, district health offices should collaborate with education offices to improve the educational status of women and men. Comprehensive nutrition education and health promotion programs in health facilities should focus on the importance of ANC follow-up and adherence to IFA tablets, which in turn will improve pregnant mothers' knowledge of anemia and the benefits of IFA tablets. Special attention should be given to young mothers under the age of 25.

### Supplementary Information

Supplementary Information.

## Data Availability

The datasets used and/or analysed during the current study are included within the manuscript and the detail are uploaded as supplementary files.
