# Author Correction: Compliance to iron folic acid supplementation and its associated factors among pregnant women attending Antenatal clinic in Wondo district: a cross-sectional study

**DOI:** 10.1038/s41598-024-52765-2

**Published:** 2024-01-26

**Authors:** Taye Mengistu, Bikila Lencha, Ashenafi Mekonnen, Sisay Degno, Daniel Yohannis, Girma Beressa

**Affiliations:** 1 Wondo District Health Office, Wondo West Arsi Zone, Oromia, Ethiopia; 2 Department of Public Health, Madda Walabu University, Shashemene, Oromia Ethiopia; 3 Department of Midwifery, Madda Walabu University, Shashemene, Oromia Ethiopia; 4 Department of Public Health, Madda Walabu University, Bale Goba, Oromia Ethiopia

Correction to: *Scientific Reports* 10.1038/s41598-023-44577-7, published online 14 October 2023

The original version of this Article contained an error in the order of the author names, which was incorrectly given as Bikila Lencha, Taye Mengistu, Ashenafi Mekonnen, Sisay Degno, Daniel Yohannis and Girma Beressa.

The original Article has been corrected.

