# Supplementary material for: Compliance to iron folic acid supplementation and its associated factors among pregnant women attending Antenatal clinic in Wondo district: a cross-sectional study
Source: Sci Rep. 2023 Oct 14;13:17468. doi: 10.1038/s41598-023-44577-7 (PMC10576767; doi:10.1038/s41598-023-44577-7)
Supplement: Supplementary file 1 — Supplementary Information. [file 41598_2023_44577_MOESM1_ESM.pdf]

Compliance to IFA and associated factors among pregnant women in Wondo district, West Arsi zone, Oromia Regional state, August, 2019.

Part I: Socio-economic and Demographic characteristics of participants

**S.N**	**Question**	**Response Categories**	**Skip**
**101**	What is your age? (in years)	_________years	
**102**	What is your current marital status?	1. Single<br>2. Married<br>3. Divorced<br>4. Widowed	
**103**	What is the religion you follow?	1. Orthodox<br>2. Protestant<br>3. Muslim<br>4. Others (specify)__________	
**104**	Where is your current place of<br>Residence?	1. Urban<br>2. Rural	
**105**	How many are your total family size?	_____in Numbers	
**106**	What is your level of education?	1. No formal education<br>2. Primary (grade 1-8)<br>3. Secondary and above	
**107**	What is your current Occupation?	1. Housewife<br>2. Government employee<br>3. Private employee<br>4. Daily Laborer<br>5. Merchant<br>**6.**Others(Specify)__________	
**108**	What is the current occupation of your husband?	1. Farmer<br>2. Government Employee<br>3. private employee	

		4. Daily laborer<br>5. Merchant<br>**6.**Others(specify)____________	
**109**	What is the educational level of your husband?	1. No formal education<br>2. Primary (grade 1-8)<br>3. Secondary and above	
**110**	What is the average monthly income of your Family? (in Ethiopian birr)	__________**Birr**	

Part II: Pregnancy and Health Status Characteristics

**S.N**	**Question**	**Response categories**	**skip**
**201**	How many pregnancies have you had until now? (in number)	____________pregnancy	
**202**	How many deliveries have you had until now?	_____________deliveries	
**203**	Have you ever had a pregnancy that ended in stillbirth?	0. No<br>1. Yes	**0→205**
**204**	If yes, How many stillbirths did you have? In numbers	________stillbirth	
**205**	Did you ever had history of abortion?	0. No<br>1. Yes	**0→207**
**206**	If yes, How many abortions do you have? In numbers	_________Abortions	
**207**	At what gestational age did you start ANC visit?<br>(In weeks) (check card)	_____________ **Weeks**	

**208**	How many times did you receive ANC? In numbers	_____________ **Times.**	
**209**	How many weeks is your gestational age now?(check card)	_____________ **week**	
**210**	From where did you receive the first ANC service?	1. Health post<br>2. Health center<br>3. Hospital<br>4. Private clinic<br>5. Others(specify)_____	
**211**	Did you tested blood for **ANEMIA** during your first ANC visit of current pregnancy?(Check card)	0. No<br>1. Yes	0→301
**212**	If yes for **Q 211** Did you have history of Anemia?(check card)	0. No<br>1. Yes	

Part III: Knowledge of Anemia and Its Prevention

**S.N**	**Questions**	**Categories of response**	**Skip**
**301**	What sign and symptom does anemia show on pregnant women? (multiple answer is possible)	1. weakness<br>2. headache<br>3. Dizziness<br>4. palmer pallor<br>5. rapid heartbeat	
**302**	What are the causes of Anemia? (Multiple is answer possible)	1. Consuming unbalanced diet<br>2. Deficiencies of iron	

		folate<br>3. Loss of blood<br>4. Chronic infection (TB,HIV)<br>5. Malaria<br>6. Hookworm infection	
**303**	What are the consequence of Anemia? (more than one answer is possible)	1. Maternal mortality<br>2. Low birth weight<br>3. Infant mortality<br>4. Impaired development<br>5. Impaired cognition	
**304**	Who are the most susceptible groups to Anemia? (more than one answer is possible)	1. Pregnant women<br>2. Infant<br>3. Adolescent girls<br>4. Children<br>88. Don't know	
**305**	What are the prevention methods of anemia? (more than one answer is possible)	1. Supplementation with IFA<br>tablet<br>2. Consumption of food rich<br>in dietary Iron/ folate<br>3. De-worming during ANC<br>4. Malaria treatment and using bed net	

Part IV: Knowledge on Iron folic acid Supplement

S.N	Question	Response categories	Skip
**401**	What is its benefit of taking Iron folic acid supplement? (more than one answer is possible)	1. Prevent Maternal Death<br>2. Prevent infant mortality<br>3. Prevent Birth defects<br>4. prevent anemia<br>5.gives strength for mothers	
**402**	At what frequency does iron folic acid tablets should have been taken during pregnancy?	1. Daily<br>2. Weekly<br>3. Twice weekly<br>4.As remembered<br>5.When I feel sick<br>88.others	
**403**	For how many days iron folic acid supplement should be taken during Pregnancy?	_____________days	
**404**	What are the possible side effects of iron folic acid supplement? (Multiple answers possible)	1. poor appetite<br>2. Nausea/Vomiting<br>3. Constipation<br>4. stool discoloration<br>5. Abdominal cramps<br>6.Others	

Part V: Compliance Related Questions

S.N	Question	Response categories	Skip
**501**	For how many days you take the iron<br>folic acid tablets during your current Pregnancy?	______________ days	
**502**	How many tablets did you take	_______________ tablets	

	totally?		
**503**	How frequently did you take your iron folic acid tablet?	1. daily<br>2. Weekly<br>3.twice weekly<br>4.As remembered<br>5.When I feel sick<br>88.others	
**504**	If, your answer for Q **503** is daily How many tablet you take daily?	1-one<br>2-two<br>3-more than two<br>4-others	
**505**	How many tablets did you take in the past (7 days) one week?	______________tablets	If<4→507
**506**	If you take ≥4 tablets what motivate you to take the tablet properly?(multiple answer possible)	1. I perceive that tablet improve health.<br>2. Insistence by health worker<br>3. Family support<br>4. Belief that tablet prevents Anemia<br>5. Others (specify)__________	
**507**	If, you had taken < 4 tablets in the past<br>one week what was reason for missing<br>the tablets? (multiple answer possible)	1. Forget fullness<br>2. Frustration of too many tablets<br>3. Due to side effects<br>4. Fear of harm to fetus<br>5. Fear of overweight baby<br>6. Due to traveling to other place<br>7. Other(specify)____________	
**508**	If the answer to **Q 507** was due to	1. Poor appetite	

	side effects (choice no 3) which side<br>effect you face? (multiple answer possible)	2. Nausea/Vomiting<br>3. Constipation<br>4. stool discoloration<br>5. Abdominal cramps<br>6.Others	
**509**	What action would you take for the side effect you face?	1. Report to the health care provider<br>2. Taking supplements with meals<br>3. Keep taking the tablet<br>4. Stop taking the tablet<br>5. Others(specify)_________	

Part VI: Health facility related question

**S.N**	**Questions**	**Categories of response**	**Skip**
**601**	Is there any counseling about IFA tablet during ANC visit?	0. No<br>1. Yes	0→603
**602**	If your answer to **Q 601** is **yes**, what was the Issue?(multiple answer possible)	1. Benefits of IFAS supplements<br>2. Frequency of the supply<br>3. Duration of supply<br>4. Possible Side effect of the supplements<br>5. Management of side effects<br>6. Food source of iron/ folate.<br>7. Others(specify)____________	
**603**	How much minutes from your home to reach nearby facility?	____________minutes	
**604**	What is the average time you spent in	_____________ minutes	

	the health facility while you were collecting iron folic acid supplement?		

Thank you for your participations!
